# Direct imaging of the three-dimensional ultrastructure of neuronal organelles

**DOI:** 10.1007/s12565-025-00888-5

**Published:** 2025-08-05

**Authors:** Daisuke Koga, Ryosuke Morinaga, Satoshi Kusumi

**Affiliations:** 1 Department of Microscopic Anatomy and Cell Biology, Asahikawa Medical University, 2-1-1-1 Midorigaoka-Higashi, Asahikawa, Hokkaido 078-8510 Japan; 2 Department of Morphological Sciences, Graduate School of Medical and Dental Sciences, Kagoshima University, 8-35-1 Sakuragaoka, Kagoshima, 890-8544 Japan

**Keywords:** Osmium maceration method, Scanning electron microscopy, Three-dimensional, Organelles; neurons

## Abstract

In the context of cell morphological analyses observing organelles embedded within the cell matrix is difficult. The osmium maceration method is a unique technique used to directly observe the three-dimensional structure of organelles through scanning electron microscopy, without requiring time-consuming and labor-intensive reconstruction. In this method, tissues are immersed in a diluted osmium solution for several days to remove cytosolic soluble proteins and filamentous structures, including microfilaments, intermediate filaments, and microtubules, from the freeze-cracked surfaces of cells, leaving the subcellular structures, Golgi apparatus, mitochondria, and smooth and rough endoplasmic reticulum intact. Specimen preparation involves several key steps, specifically pre-fixation with aldehyde fixatives, tissue excision, trimming, post-fixation with osmium tetroxide solution, dimethyl sulfoxide cracking (i.e., freeze-cracking), the thawing of cracked tissues, osmium maceration, osmium fixation, conductive staining (tannin–osmium method), dehydration, drying, mounting, metal coating, and scanning electron microscopy observations. Here, we present a step-by-step protocol based on the maceration method using neural cells as an example, ensuring reproducibility and consistent results for neurons and various other cell types. Moreover, the results presented indicate that the osmium maceration method is effective for elucidating the three-dimensional intracellular ultrastructure of neurons.

**Supplementary Information:**

The online version contains supplementary material available at 10.1007/s12565-025-00888-5.

## Introduction

In contrast to the transmission electron microscopy (TEM) analysis of resin-embedded tissue sections, scanning electron microscopy (SEM) uses secondary electron signals to enable the direct three-dimensional (3D) visualization of the surface topography of tissues and cells. Using conventional specimen preparations, SEM provides valuable morphological information regarding hollow organs, such as the intestines and trachea. For instance, for intestinal specimens, the 3D structure of villi and ultrastructural details of the apical surfaces of absorptive and goblet cells, such as microvilli and secretory granules, can be clearly observed using SEM. In addition, SEM has revealed the 3D ultrastructural features of various cell types, such as podocytes in the kidney glomeruli (Takahashi-Iwanaga 2002), hair cells of the inner ear cochlea (Grillet 2022), and cells in hollow organs. As glands, blood vessels, nerves, and muscles are covered by the basal membrane and wrapped by the surrounding connective tissue, observing the surface structures of cells located in the connective tissues is difficult using conventional SEM preparations. To address this issue, connective tissue digestion methods have been established to remove the fibrous components of connective tissues and basal membrane covering the basal surfaces of cells, leaving only the cellular components (Takahashi-Iwanaga and Fujita 1986; Ushiki and Ide 1988). For SEM, the alkali-water maceration method, which removes cellular components while preserving the collagen fibrillar network, has contributed to elucidating the 3D architectures of reticular tissues, such as lymph nodes and the spleen (Ohtani 1987). Further, the vascular casting method, which involves the corrosion of tissues through a chemical process following the hardening of resin injected into blood vessels, has been employed for the 3D analysis of microcirculation in various tissue types, using SEM (Murakami 1971). Thus, specimen preparation for SEM is characterized by the selective preservation of structures of interest with the removal of other components. In contrast, attempts to observe the internal structure of cells using SEM were made in the 1970s, shortly after, it was first applied in biology. In the 1970s, various cell-cracking techniques, such as resin cracking and freeze-cracking, were introduced by electron microscopists (Tanaka et al. 1974; Tokunaga et al. 1974).

**Fig. 1:**
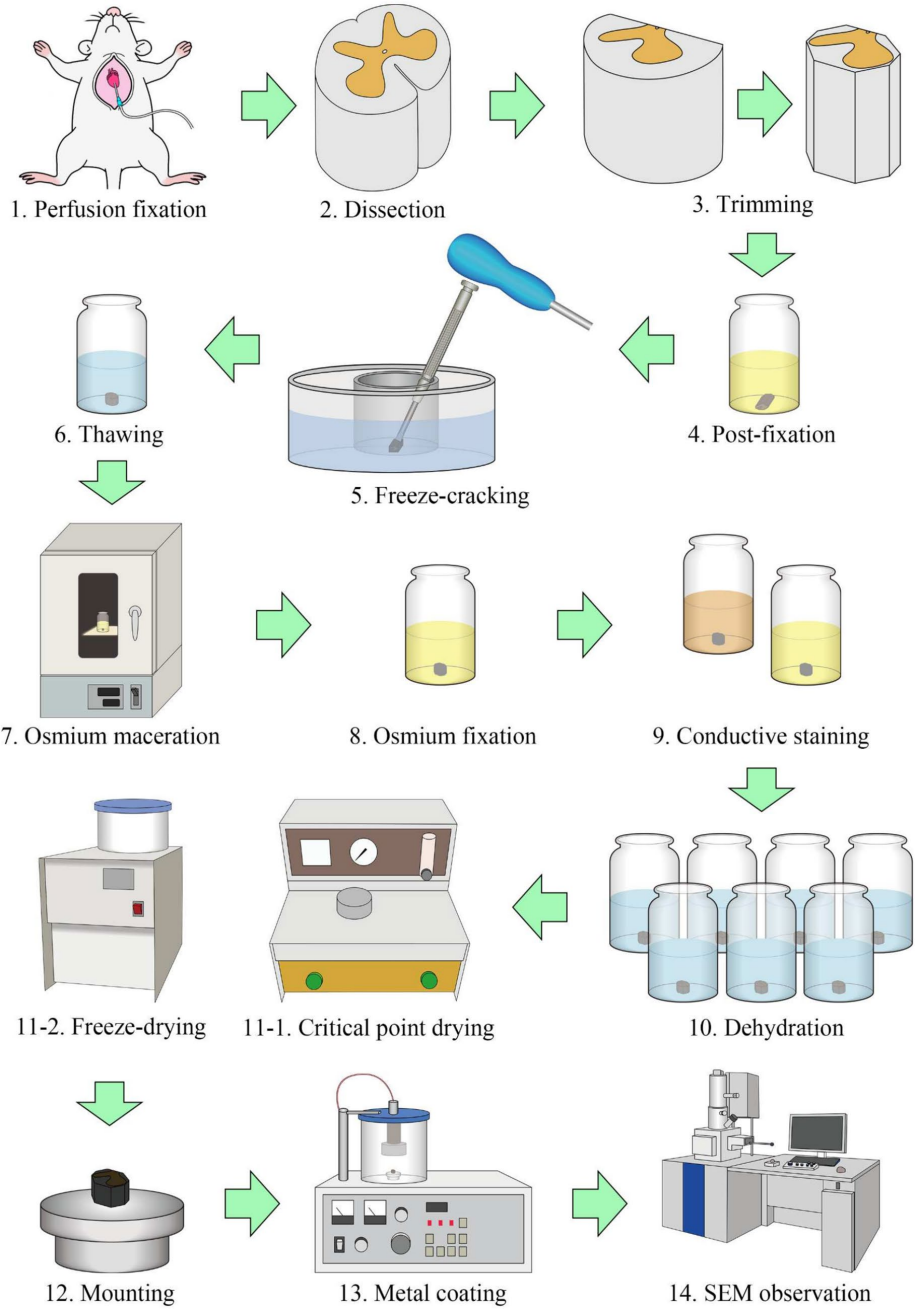
Flow diagram of the osmium maceration method

Although these methods enable the precise cleavage of cells, observing organelles embedded within the cell matrix remains difficult. Professor Keiichi Tanaka and colleagues have resolved this issue. They found that diluting osmium tetroxide to approximately one-tenth of the concentration typically used for membrane fixation induces a protein-dissolving effect. Using this diluted solution, they succeeded in removing soluble cytosolic proteins and filamentous structures, such as actin, intermediate filaments, and microtubules, from the cracked cell surfaces. This method, termed the osmium maceration method, is the only technique that enables the direct 3D visualization of cell organelles via SEM (Tanaka and Naguro 1981). Originally designed to target tissues fixed by osmium tetroxide alone, through immersion, this method was later applied to tissues fixed through perfusion with aldehyde fixatives (Tanaka and Mitsushima 1984). This modification facilitates the application of this method to neural tissues that are highly susceptible to post-mortem changes. In this study, we introduce the osmium maceration method with an example using neural tissues from the trigeminal ganglia and spinal cords (Fig. 1).

## Protocols

### Materials

The key resources are listed in the table below.

**Table:** 

Reagent or Resource	Source	Identifier
*Chemicals and Reagents*		
Sodium Chloride	Nacalai Tesque	31,320-05
Sodium Dihydrogenphosphate Dihydrate	Nacalai Tesque	31,718-15
di-Sodium Hydrogenphosphate 12-Water	Nacalai Tesque	31,723-35
Paraformaldehyde	Merck	30,525-89-4
Glutaraldehyde (25% in water)	Nacalai Tesque	17,003-05
Osmium tetroxide crystals	Nisshin EM	Cat.No.300
Dimethyl Sulfoxide (DMSO)	Wako	043-07216
Tannic Acid	Nacalai Tesque	32,616-02
Ethanol (99.5)	Nacalai Tesque	14,713-53
3-Methylbutyl Acetate (Isopentyl Acetate)	Nacalai Tesque	02710-95
t-Butyl Alcohol (2-Methyl-2-propanol)	Nacalai Tesque	06104-25
ThreeBond 3350C	Nisshin EM	Cat.No.713
*Sample Preparation Materials*		
SEM Cylinder Specimen Mounts	Nisshin EM	Cat.No. S-HM φ15 × 7 M4 made of aluminum
Conductive carbon double-sided tape	Nisshin EM	Cat.No. 7321
*Equipment*		
Temperature-controlled chamber	AS ONE	FCI-280 Cool Incubator
Critical point dryer	Hitachi	HCP-2
Freeze-drying device	EIKO	ID-2
Ion-sputter coater	Hitachi	E-1030
Dissecting microscope	Leica	S6E LED 2500
Scanning electron microscope	Hitachi	Regulus 8220

## Methods

Male Wistar rats, purchased at 8 weeks of age from Sankyo Laboratory Service Co., Ltd. (Tokyo, Japan) were used in this study. Animal experiments were approved by the Committee of Ethics on Animal Experiments at Asahikawa Medical University and conducted according to the Asahikawa Medical University Animal Experimentation Regulations (Permission number: R6-103-2, R6-104).

### Step 1: Fixation of tissues: perfusion fixation

Perfusion fixation is useful for preserving the ultrastructure of nerve tissues, which are prone to post-mortem degeneration. This section briefly describes the perfusion fixation preparation using rats. For details of the procedure, standard textbooks should be consulted or the procedure should be performed according to the established protocols of each department.

Before starting perfusion fixation, the following materials should be prepared:

- A fixative with a final concentration of 0.5% glutaraldehyde and 0.5% paraformaldehyde in 0.1 M phosphate buffer (pH 7.2–7.4; 1,000 mL/kg BW).

**Note**: Preparing an 8% paraformaldehyde stock solution in advance is recommended. The solution should be stored in a refrigerator and will remain stable for approximately 1 month.

- Physiological saline solution (500 mL/kg BW).

**Note:** The fixative (0.5% glutaraldehyde and 0.5% paraformaldehyde in 0.1 M phosphate buffer, pH 7.2–7.4) and physiological saline solution for flushing out the blood should be prepared immediately before use and kept at room temperature (20–25℃).

- A mixture of ketamine (75 mg/kg BW) and xylazine (10 mg/kg BW) is used as the anesthetic agent and is intraperitoneally administered.
- Forceps, scissors, bone nipper, mosquito forceps, Bulldog clamp forceps, razor blades.

- Inject a mixture of ketamine (75 mg/kg BW) and xylazine (10 mg/kg BW) intraperitoneally into rats to induce deep anesthesia.
- After making an incision in the thoracic skin, cut through the costal cartilage and thoracic muscles, including the deep pectoral and external intercostal muscles, to expose the heart within the thoracic cavity.
- Insert the perfusion cannula into the left ventricle, advance its tip to the aortic arch, and secure both the cannula and arch using forceps.
- Cut the right atrial appendage with scissors.
- Perfuse the rats via the vascular system with physiological saline (500 mL/kg BW) at room temperature (20–25 °C) at a rate of 100–200 mL per 10 min to flush out the blood.
- Switch to perfusion with a mixture of 0.5% paraformaldehyde and 0.5% glutaraldehyde in 0.1 M phosphate buffer (pH 7.2–7.4; 1,000 mL/kg BW), maintaining the same flow rate as that used for the physiological saline.
- Stop the perfusate flow, withdraw the perfusion cannula from the left ventricle, and proceed with tissue sampling.

### Step 2: Excision of tissues: spinal cords and trigeminal ganglia

**Fig. 2:**
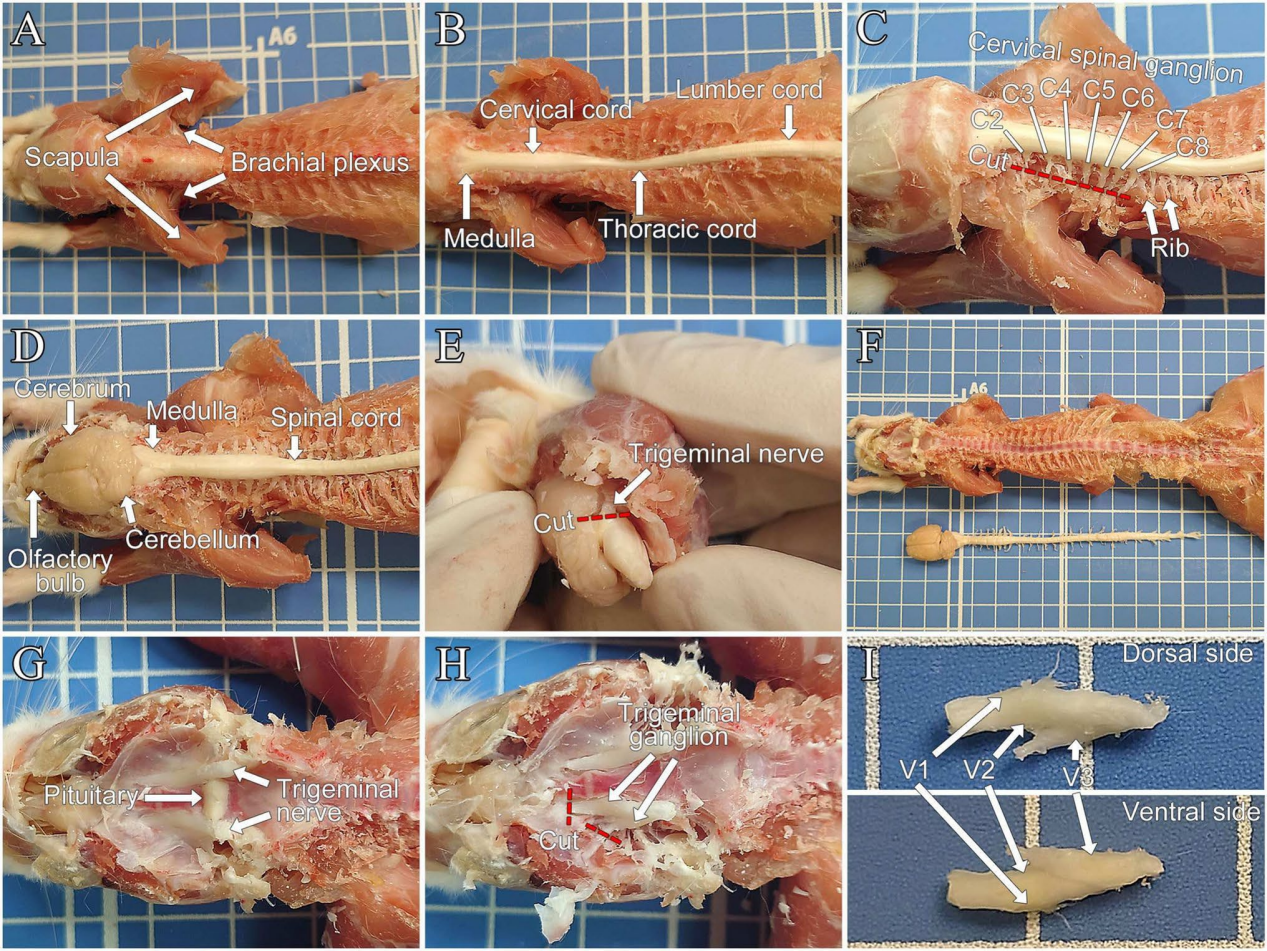
**A** Dorsal surface of the skull and vertebrae. **B** Dorsal surface of the spinal cord. **C** Position of the cervical spinal ganglion. The dorsal root ganglia (C1–C8) are located at the bases of the dorsal roots emerging bilaterally from the spinal cord. The dorsal root distal to the ganglion is cut (red dotted line). **D** Dorsal surface of the brain and spinal cord. **E** Ventrolateral surface of the brain. Thick trigeminal nerves emerge bilaterally from the ventrolateral surface of the pons. The nerves at their proximal origin near the brain are cut (red dotted line). **F** Whole brain and spinal cord. **G** Internal base of the skull after brain removal. **H** Position of the trigeminal ganglion. The nerves distal to the ganglion are cut (red dotted line). **I** Trigeminal ganglion. The trigeminal ganglion divides into three branches: the ophthalmic nerve (V1), maxillary nerve (V2), and mandibular nerve (V3). The ganglia originating from each nerve branch are indicated with arrows
- Remove the dorsal vertebral arch to expose the dorsal surface of the spinal cord (Fig. 2B).
- Remove the transverse processes and lateral portions of the vertebral arches to expose the dorsal roots.
- Cut the dorsal root distal to the ganglia to collect the spinal cord with the dorsal root attached (Fig. 2C).
- Remove the dorsal portion of the skull, leaving only the cranial base, including the sphenoid bone and basilar part of the occipital bone, to expose the brain (Fig. 2D).
- Cut the trigeminal nerves at their proximal origin on the ventrolateral surface of the pons (Fig. 2E).
- Extract the brain and spinal cord with the dorsal roots attached from the vertebral column (Fig. 2F).
- After extracting the pituitary gland, remove the cranial base and dura mater around the trigeminal ganglia (Fig. 2G).
- Sever the trigeminal nerves distal to the trigeminal ganglia (Fig. 2H).
- Extract the trigeminal ganglia (Fig. 2I).

The following materials should be prepared before beginning the excision of tissues:

- Fine forceps, scissors, and bone nipper.

- Remove the skin on the back to expose the superficial dorsal muscles, including the trapezius, splenius, and erector spinae.
- Cut along the midline through the muscles connecting the scapula to the spine, such as the trapezius and rhomboid, and reflect them laterally. Then, remove the dorsal muscles, including the splenius, serratus dorsalis, erector spinae, along with spinous processes of the vertebrae to expose the dorsal vertebral arches (Fig. 2A).

### Step 3. Trimming tissues

Before trimming the tissues, the following materials should be prepared.

- A 0.1 M phosphate buffer solution (pH 7.2–7.4.)
- Fine forceps, fresh razor blades, and glass scintillation vials (15 mL).

- Cut the excised tissues into small pieces and trim tissues to approximately 2 × 2 × 5 mm using razor blades.

**Critical**: The spinal cord should be trimmed to remove the maximum amount of white matter. White matter interferes with the immersion of the osmium solution used as a post-fixative and prevents osmium fixation of neurons in the gray matter.

b. Place the trimmed tissues in glass vials containing 0.1 M phosphate buffer (pH 7.2–7.4) at 4 °C.

**Note:** Tissues in the buffer solution can be stored for several days in a refrigerator at 4 °C.

### Step 4. Post-fixation

Post-fixation with osmium tetroxide stabilizes the membranous structures, including the Golgi apparatus, endoplasmic reticulum (ER), and mitochondria, by fixing phospholipids.

- Prepare the post-fixative with a final concentration of 1% osmium tetroxide in 0.1 M phosphate buffer (pH 7.2–7.4).

**Note:** The osmium tetroxide crystals are dissolved in advance to prepare a 4% osmium tetroxide aqueous solution, stored in an amber bottle, and kept in a refrigerator. These crystals are poorly soluble in water and should be dissolved in advance. When osmium tetroxide crystals are dissolved in distilled water, the use of a benchtop ultrasonic cleaner facilitates efficient dissolution, which is typically completed within 2–3 h. The osmium tetroxide solution should also be handled in a draft chamber. Gloves should always be worn when handling the solution. Waste solutions must be collected in designated waste containers and disposed of in accordance with the relevant regulations of the university or research institution.

b. Remove the 0.1 M phosphate buffer from the glass vials containing the tissues, add 1% osmium tetroxide solution and fix the tissues at 4 °C on a rotator for 3 h.

**Critical**: The use of a rotator or agitator is recommended for the osmium fixation process because the penetration of osmium tetroxide into tissues is poor and requires time.

c. Tissues should be rinsed with a 0.1 M phosphate buffer solution (pH 7.2–7.4) for 10 min. Repeat this step six times (for a total of 60 min).

**Critical**: The dimethyl sulfoxide (DMSO) solution used in the next step immediately blackens when tissues with residual osmium are immersed, leading to specimen contamination.

### Step 5. DMSO cracking

Freeze-cracking with DMSO, which prevents ice crystal formation in cells, is used to expose the intracellular structures. The authors regularly employ a freeze-cracking apparatus assembled in-house. The Eiko TF-1 or TF-2 (Eiko Engineering Co., Ltd., Tokyo) is commonly used for tissue cracking (Tanaka and Naguro 1981; Tanaka and Mitsushima 1984).

**Fig. 3:**
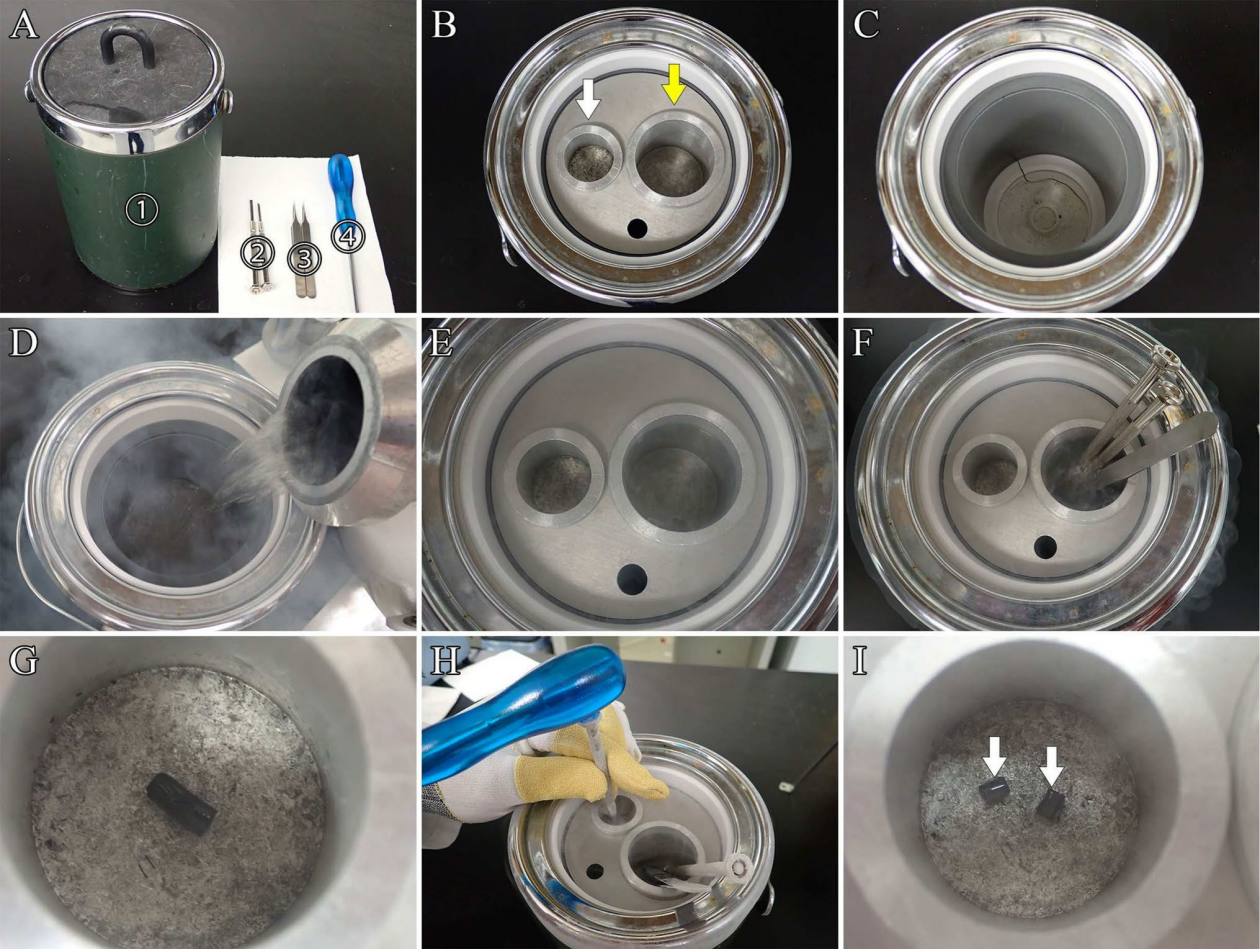
Freeze-cracking procedure. **A** ① Freeze cracking apparatus, ② precision flathead screwdrivers, ③ fine forceps, and ④ screwdriver. **B** Freeze-cracking apparatus. White arrow: aluminum plate used for freeze-cracking specimens; yellow arrow: aluminum container for chilling precision flathead screwdrivers and fine forceps. **C** Interior of the dewar in the freeze-cracking apparatus. **D** Liquid nitrogen is poured into the Dewar freeze-cracking apparatus. **E** The aluminum plate and aluminum container used to chill the precision flathead screwdrivers and fine forceps are cooled with liquid nitrogen. **F** Liquid nitrogen is added to the aluminum container, and the screwdrivers and forceps are cooled. **G** Specimens are placed on an aluminum plate and cooled. **H** The specimens are freeze-cracked and split into two pieces using fine forceps and a hammer. **I** Freeze-cracked specimens (arrows)
- Fine forceps, precision flathead screwdrivers, and screwdriver (Fig. 3A).
- 25% and 50% DMSO solutions in distilled water.

**Note:** Since dissolving DMSO in water generates heat, the solution should be cooled before use. Gloves should always be worn when handling the solution. Waste solutions must be collected in designated waste containers and disposed of in accordance with the relevant regulations of the university or research institution.

Before this step, the following materials should be prepared:

- Liquid nitrogen.
- Freeze-cracking apparatus (custom-made device; Fig. 3A) or Eiko TF-1 or TF-2 (commercially available devices).

- Immerse specimens in 25% and 50% DMSO solutions for 30 min each. Fill the glass vials containing approximately four tissue blocks with 10 mL of DMSO solution.

**Critical**: The use of a rotator or agitator is recommended to ensure that the DMSO solution penetrates the tissues.

b. Continue pouring the liquid nitrogen into the dewar of the freeze-cracking apparatus until it reaches the temperature of liquid nitrogen (Fig. 3D).
c. Place the aluminum container used for chilling the fine forceps, precision flathead screwdrivers, and aluminum plate used for cooling the tissues, into the freeze-cracking apparatus and wait until they reach the temperature of the liquid nitrogen (Fig. 3E).
d. Chill the fine forceps and precision flathead screwdrivers in an aluminum container containing liquid nitrogen in the freeze-cracking apparatus (Fig. 3F).
e. Place the tissues on the aluminum plate, pre-cooled with liquid nitrogen, into the freeze-cracking apparatus and allow the tissues to stand for 3 min (Fig. 3G).
f. Crack the tissues into two pieces using a precision flathead screwdriver, pre-chilled with liquid nitrogen, and a hammer (Fig. 3H, I).

**Note:** The handle of the screwdriver is used as a hammer. Cryogenic gloves are essential to ensure safety and prevent frostbite when handling liquid nitrogen.

### Step 6. Thawing

- Collect freeze-cracked tissues using fine forceps pre-cooled with liquid nitrogen, place them into a 50% DMSO solution at room temperature, and incubate them for 5 min until the tissues are completely thawed.
- Rinse the freeze-cracked tissues with a 0.1 M phosphate buffer solution (pH 7.2–7.4) for 10 min. Repeat this step six times (for a total of 60 min) to completely remove the DMSO from the tissues.

**Critical**: If any residual DMSO remains in the tissue, the osmium maceration solution in the following step will immediately darken upon specimen immersion, leading to contamination.

### Step 7. Osmium maceration procedure

During this process, the cytoplasmic matrix is removed from the cracked cell surfaces, whereas the membranous structures remain intact.

**Fig. 4:**
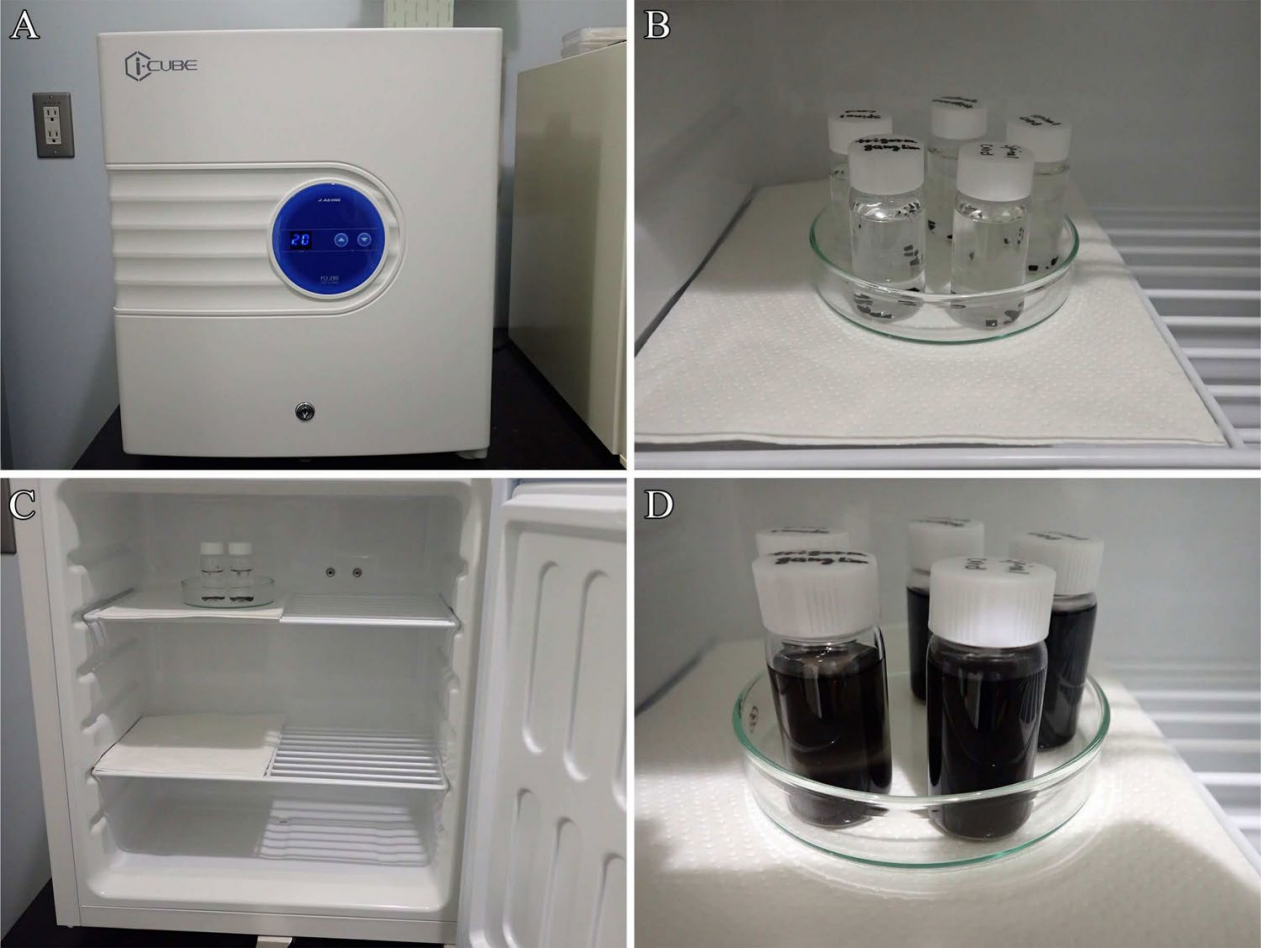
Osmium maceration procedure. **A** Temperature-controlled chamber (FCI-280 Cool Incubator; AS ONE). **B** Specimens in the osmium maceration solution. **C** Vials containing specimens and osmium maceration solution are placed in a temperature-controlled chamber maintained at 20 °C for 120 h. **D** Vials containing specimens and osmium maceration solution after the osmium maceration procedure. Note the blackened osmium maceration solution

Before this step, the following materials and devices should be prepared:

- The osmium maceration solution at a final concentration of 0.1% osmium tetroxide in 0.1 M phosphate buffer (pH 7.2–7.4).
- Temperature-controlled chamber (Fig. 4A).

- Immerse four tissue blocks in vials containing 10 mL of the osmium maceration solution, place them in a temperature-controlled chamber at 20 °C, and incubate them for 120 h (Fig. 4B, C).
- After the maceration, check that the osmium maceration solution has blackened (Fig. 4D).

**Note:** The optimal duration of the osmium maceration procedure, to completely remove the cytoplasmic matrix from the cracked surfaces of cells, varies depending on the cell type. Generally, maceration for 120 h is necessary to expose the 3D ultrastructure of organelles in various cell types.

**Critical**: To determine the appropriate osmium maceration time for specimen preparation, various maceration durations, including 48, 72, 96, and 120 h, are tested using specimens examined for the first time, and the optimal time is selected. Insufficient maceration results in residual cytosolic materials remaining on the surface of organelles, which obscure their ultrastructure and hinder detailed observation of membranous organelles (Koga et al. 2020).

**Note:** Osmium tetroxide solution should be handled in a fume hood. Always wear gloves when handling the solution. The waste solution must be stored in a designated waste container.

### Step 8. Osmium fixation

Osmium treatment halts the progression of specimen maceration, effectively terminating the process.

- Prepare 1% osmium tetroxide in 0.1 M phosphate buffer (pH 7.2–7.4).
- Remove the osmium maceration solution from the vials containing osmium-macerated tissues, add 1% osmium tetroxide solution, and place the sample at 4 °C for 1 h. Use 2 mL of 1% osmium tetroxide for four tissue blocks.
- Rinse the tissues with a 0.1 M phosphate buffer solution (pH 7.2–7.4) for 10 min. Repeat this step six times for a total of 60 min.

**Note**: Osmium tetroxide solution should be handled in a fume hood. Always wear gloves when handling the solution. The waste solution must be stored in a designated waste container.

**Critical**: Tissues should be thoroughly rinsed with the buffer solution. Residual osmium in the specimens binds to the tannic acid used in subsequent treatments, resulting in precipitate formation and sample contamination.

### Step 9. Conductive staining

This process imparts conductivity to non-conductive bio-specimens.

- Prepare a 1% tannic acid solution in 0.1 M phosphate buffer (pH 7.2–7.4).

**Critical**: A tannic acid solution should be prepared immediately prior to use.

b. Treat the tissues with a 1% tannic acid solution for 1 h at room temperature. Use 10 mL of the tannic acid solution for four tissue blocks.

**Note**: Tannic acid is used as a mordant to effectively facilitate the binding of metals, such as osmium, to the specimens.

c. Rinse the tissues with a 0.1 M phosphate buffer solution (pH 7.2–7.4) for 10 min. Repeat this step six times, for a total of 60 min.

**Critical**: Residual tannic acid in the tissue should be completely removed by rinsing the sample with a buffer solution, as it binds to osmium during the subsequent osmium treatment, potentially contaminating samples; moreover, granular deposits adhere to the specimen surface.

d. Prepare 1% osmium tetroxide in a 0.1 M phosphate buffer (pH 7.2–7.4).
e. Treat the tissue blocks with a 1% osmium tetroxide solution at 4 °C for 1 h. Use 2 mL of the osmium tetroxide solution for four tissue blocks.

**Note:** Osmium tetroxide solution should be handled in a fume hood. Always wear gloves when handling the solution. The waste solution must be stored in a designated waste container.

### Step 10. Dehydration

SEM specimens are generally dried using critical-point drying or freeze-drying. As a preceding step in the drying process, the specimens are dehydrated with ethanol.

- Prepare 70, 80, 90, 95, and 100% ethanol.

**Note:** Each concentration of ethanol is prepared through dilution using 100% ethanol.

b. Dehydrate the tissues through a graded ethanol series (70, 80, 90, 95, and 100% ethanol), with each step lasting for 15 min. Use 10 mL of each ethanol solution for four tissue blocks.

### Step 11. Drying

SEM specimens are dried using critical-point drying or freeze-drying methods.

#### 11-1. Critical point drying

Critical-point drying using liquid carbon dioxide is commonly employed. The specimens are subjected to solvent exchange from ethanol to isoamyl acetate, then substituted with liquid carbon dioxide in the pressure vessel of a critical-point dryer and dried under conditions exceeding the critical pressure (72.8 kg/cm^2^) and temperature (31 °C) of carbon dioxide (Flegler et al. 1993), with gases exhausted from the vessel.

**Fig. 5:**
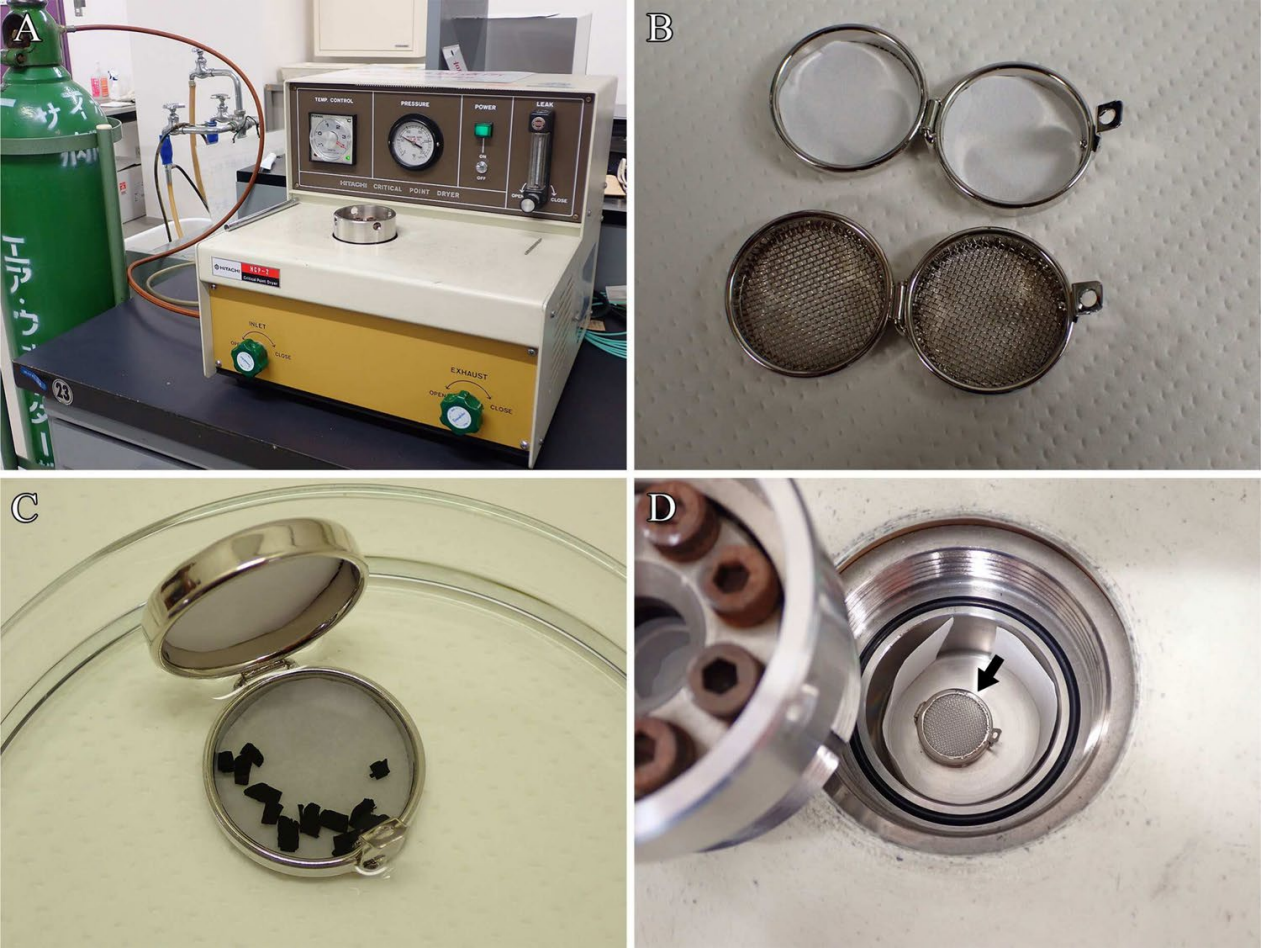
Critical-point drying. **A** Critical point dryer (HCP-2; Hitachi). **B** Specimen baskets: filter paper is used to cover both the top and bottom inner surfaces of each basket. **C** After substituting the dehydrated specimens with isoamyl acetate, specimens are placed in baskets. **D** The basket (arrow) is transferred to the pressure vessel of the critical-point dryer and lid of the vessel is closed

Before this step, prepare the following materials and devices:

- Isoamyl acetate.
- Critical-point dryer (Fig. 5A).

- After dehydration with ethanol, substitute the specimens with isoamyl acetate for 5 min. Repeat this step twice, for a total of 10 min. Use approximately 5 mL of isoamyl acetate for four tissue blocks.

**Note:** Isoamyl acetate is used as an intermediate solution between ethanol and liquid carbon dioxide. Isoamyl acetate should be handled in a fume hood. Waste solutions must be collected in designated waste containers and disposed of in accordance with the relevant regulations of the university or research institution.

b. Cut the filter paper into a round shape to fit the diameter of the specimen basket for critical-point drying and use the filter paper to cover both the top and bottom inner surfaces of the basket (Fig. 5B).

**Note:** The baskets are kept clean to prevent specimen contamination. Filter papers covering both the top and bottom inner surfaces help to prevent specimen contamination from the carbon dioxide fluid containing iron powder flowing from the gas cylinder, in addition to preventing small specimens from dispersing from the baskets.

c. Place the specimens in the specimen basket (Fig. 5C) and gently blot any excess isoamyl acetate adhering to the filter paper.
d. Quickly transfer the basket with specimens to the pressure vessel of the critical-point dryer (Fig. 5D) and close the lid of the vessel.

**Critical**: Transferring the specimens to the vessel, closing the lid, and introducing liquid carbon dioxide should be performed as quickly to avoid air-drying of the specimens.

e. Start the critical-point dryer.

**Note:** For detailed instructions on critical-point dryer use, refer to the user manual of the equipment being used.

#### 11–2. Freeze-drying

Freeze-drying removes moisture and solvents from the specimens through sublimation. To achieve this, frozen tissues are placed in a vacuum environment and maintained in a frozen state, allowing the moisture and solvents to sublimate.

**Fig. 6:**
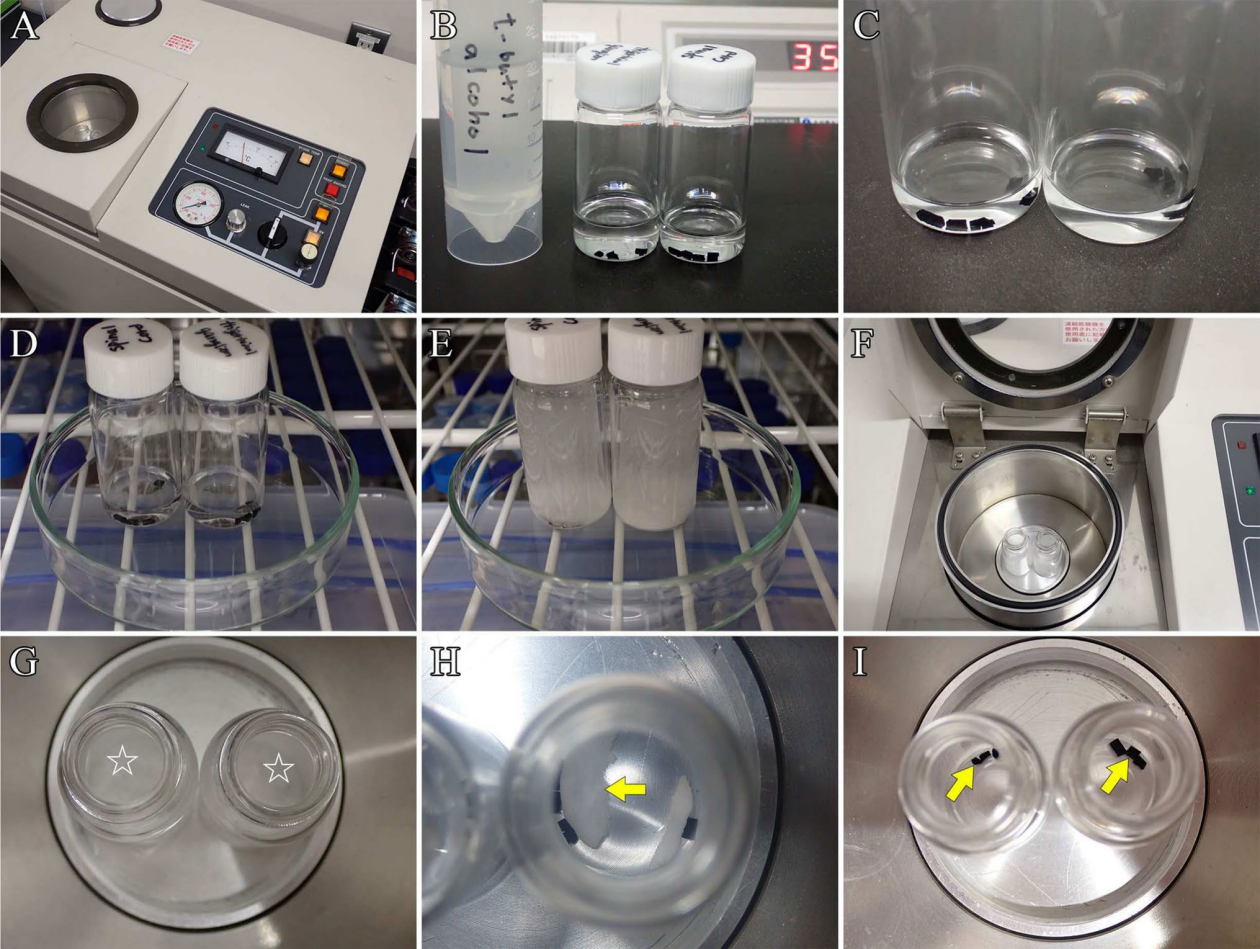
Freeze-drying. **A** Freeze-dryer (ID-2; Eiko). **B** Vials containing specimens and t-butyl alcohol are placed on a hot plate maintained at 35 °C during the substitution process. **C** The final volume of t-butyl alcohol should be just enough to slightly submerge the specimens. **D** Vials are placed in a refrigerator to freeze the specimens and t-butyl alcohol. **E** After placing the vials in the refrigerator, the specimens and solvent freeze within several minutes. **F** Vials are transferred to the specimen chamber of the freeze dryer. **G** Specimens during freeze-drying. Stars indicate frozen t-butyl alcohol. **H** One hour into the freeze-drying process. The arrow indicates residual frozen t-butyl alcohol. **I** Two hours into the freeze-drying process. T-butyl alcohol has completely sublimed. Arrows show dried specimens

Before this step, prepare the following materials and devices:

- t-Butyl alcohol.
- Freeze-dryer (Fig. 6A).

- After dehydration with ethanol, substitute the specimens with t-butyl alcohol for 10 min. This step is triplicated (for a total of 30 min). Use approximately 2–3 mL of isoamyl acetate for four tissue blocks.

**Critical**: Insufficient substitution may leave ethanol in the tissues, preventing proper drying. The substitution of ethanol with t-butyl alcohol in tissues should be performed with an adequate number of repetitions and duration.

**Note:** The freezing point of t-butyl alcohol is high (25.7 °C; Akahori et al. 1988), and tissues substituted with this solvent can be frozen in a refrigerator; t-butyl alcohol ensures excellent freeze-drying. Vials containing specimens and t-butyl alcohol are placed on a hot plate at 35 °C during the substitution to prevent the solvent from freezing (Fig. 6B). Waste solutions must be collected in designated waste containers and disposed of in accordance with the relevant regulations of the university or research institution.

b. The final amount of solvent should be sufficient to barely submerge the specimens in t-butyl alcohol, as an excessive solvent volume can lead to prolonged sublimation (Fig. 6C).

- Place the vials containing specimens and t-butyl alcohol in a refrigerator to freeze both the specimens and solvent. Both the tissues and t-butyl alcohol freeze within several minutes (Fig. 6D–E).

**Note:** Frozen t-butyl alcohol forms needle-like crystals, which cause cloudiness; however, no ice crystal damage will be observed and the fine structure of the specimens will remain well-preserved.

c. Transfer vials containing frozen tissues and t-butyl alcohol to the freeze-dryer specimen chamber (Fig. 6F).
d. Start the freeze-dryer: evacuate the vacuum in the chamber (Fig. 6G-I).

**Note:** For detailed instructions on using the freeze-dryer, refer to the user manual of the equipment being used.

### Step 12. Mounting

Process for specimen adhesion to specimen mounts.

**Fig. 7:**
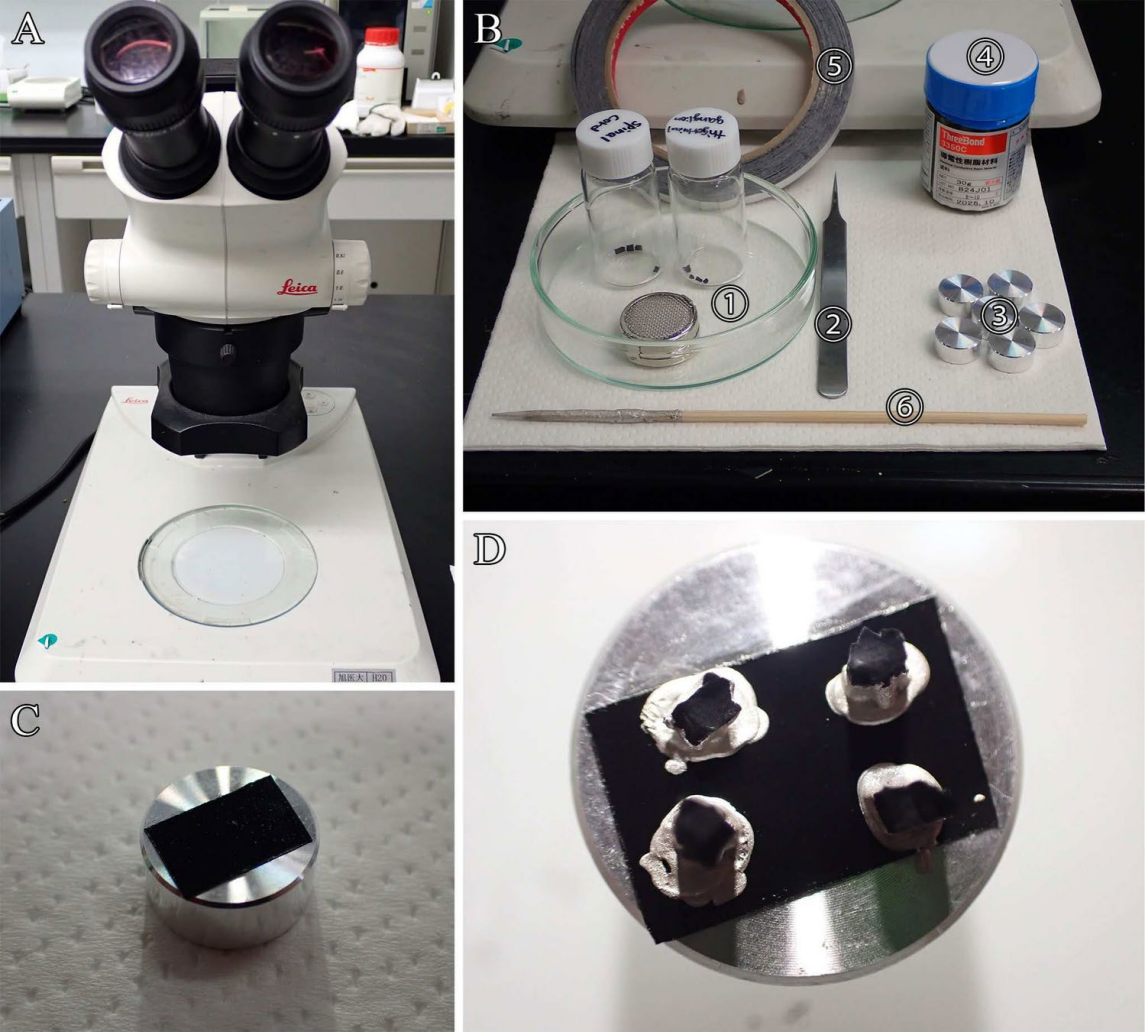
Mounting process. **A** Dissecting microscope (S6E LED 2500; Leica), **B** ① Dried specimens, ② fine forceps, ③ specimen mounts, ④ silver paste, ⑤ carbon double-sided tape, ⑥ bamboo skewer. **C** Double-sided conductive tape is attached to the mounts. **D** Specimens are mounted on carbon tape and silver paste is applied to ensure adhesion
- Carbon double-sided tape and conductive adhesives, such as silver paste (Fig. 7B).
- SEM specimen mounts (Fig. 7B).
- Fine forceps (Fig. 7B).
- Bamboo skewer (Fig. 7B).

Before this step, the following materials and devices should be prepared:

- Dissecting microscope (Fig. 7A).

- Attach double-sided conductive tape to the mounts (Fig. 7C).
- Check the observation surfaces (cracked surfaces) of osmium-macerated tissues using a dissecting microscope.
- Mount the tissues onto the carbon tape attached to specimen mounts to adhere the samples to mounts.
- Coat specimens, excluding the surfaces to be observed, with silver paste using a bamboo skewer to ensure adhesion between the specimens and mounts (Fig. 7D).

**Critical**: The gas-generation rate within the SEM specimen chamber is proportional to the number of specimens on the specimen mounts and amount of conductive adhesive, resulting in specimen contamination and SEM chamber. Therefore, the use of conductive adhesives should be minimized. The silver paste covering the specimens is sufficiently dried to progress to the next step, metal coating.

### Step 13. Metal coating

Metal coating is the process whereby metals are coated onto specimens to prevent charging artifacts and improve the efficiency of secondary electron signal generation.

**Fig. 8:**
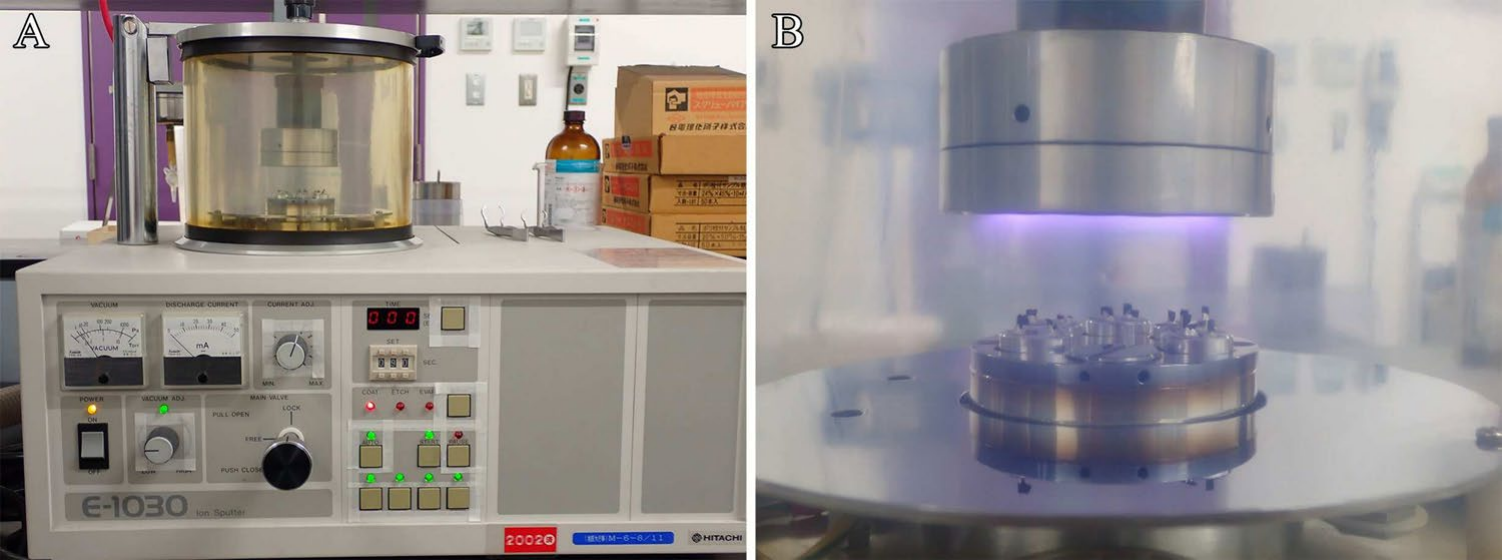
Metal coating process. **A** Ion sputter coater instrument (E1030; Hitachi). **B** Specimens are coated with 2–3 nm of platinum–palladium

**Note:** For detailed instructions on ion sputtering device use, refer to the user manual of the equipment being used.

- Specimen surfaces are coated with metals using an ion sputtering device (Fig. 8).

**Critical**: High-magnification imaging is required for the SEM analysis of osmium-macerated specimens; therefore, metal targets with fine metal particles (such as platinum and platinum–palladium alloys) should be used for metal coating. The thickness of the metal coating should be set to several nanometers, as the thick coating obscures the fine structures of the organelles and results in the observation of masses of metal particles on the specimens via SEM.

### Step 14. SEM observation

**Fig. 9:**
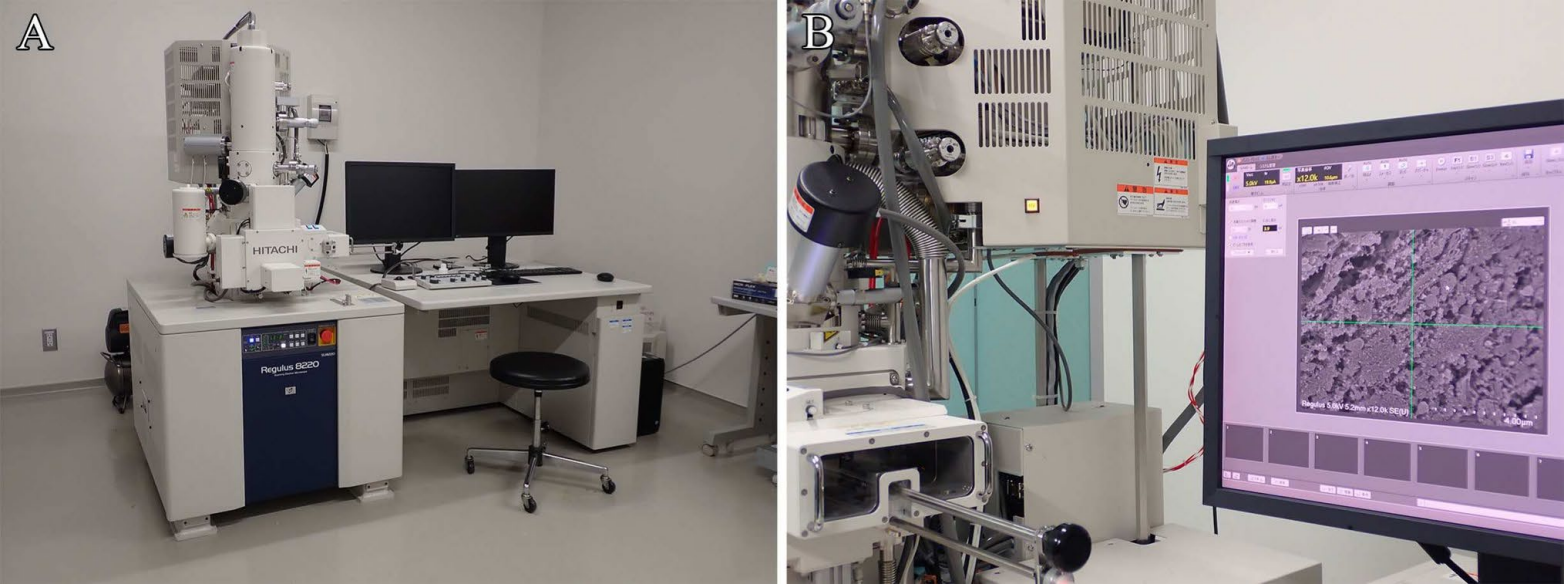
Scanning electron microscopy of osmium-macerated tissues. **A** Semi-in-lens-type field emission scanning electron microscope (Regulus; Hitachi). **B** Specimens are observed using the ultra-high resolution scanning electron microscope

The osmium-macerated tissues are observed using a high-resolution scanning electron microscope equipped with a field emission gun (Fig. 9).

## Results

**Fig. 10:**
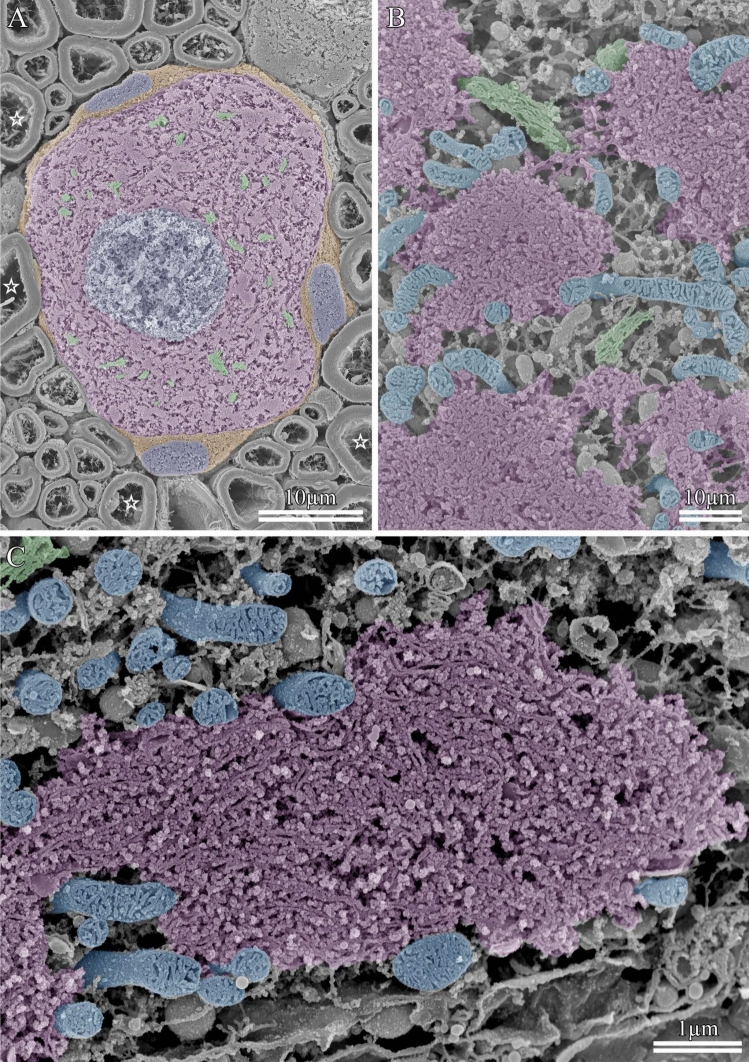
Scanning electron microscopy images of trigeminal ganglion cells and a motor neuron. **A** Overview of the soma in a trigeminal ganglion cell. Blue: nuclei, Green: Golgi apparatus, Brown: satellite cells, Magenta: soma. ☆: myelinated axons. **B** High-magnification image of organelles in the trigeminal cell. Magenta: Nissl bodies, Blue: mitochondria, Green: Golgi apparatus. **C** High-magnification image of membranous organelles in the soma of a motor neuron. Magenta: Nissl body, Green: Golgi apparatus, Blue: mitochondria

When the cracked surface of cells not subjected to the osmium maceration procedure is observed by SEM, the 3D ultrastructure of membranous organelles is not clearly visualized, as soluble cytoplasmic proteins remain largely within the cells (SI Fig. 1). SEM of osmium-macerated tissues provides information on the 3D ultrastructure of organelles, such as the Golgi apparatus, mitochondria, and ER. Figure 10A shows a low-magnification image of the freeze-cracked surfaces of trigeminal ganglion cells prepared using the osmium maceration method. Satellite cells wrapping around the somata of the ganglion cells can be observed, with myelinated axons densely surrounding the somata in the trigeminal ganglion. The nucleus is centrally located within the soma, and stacks of the Golgi cisternae are scattered throughout the cytoplasm. High-resolution SEM has provided 3D ultrastructural insights into the mitochondria, Golgi apparatus, and rough ER (Nissl bodies) in the trigeminal ganglion cells (Fig. 10B). The spatial relationships between Golgi stacks, mitochondria, and Nissl bodies are clearly visible using SEM. Figure 10C shows high-magnification images of the Golgi apparatus, mitochondria, and Nissl body in a motor neuron of the spinal cord. The 3D ultrastructure of these organelles can be visualized using SEM. Nissl bodies can be identified as densely packed regions of rough ER. Therefore, the osmium maceration method is an effective approach for elucidating the 3D intracellular ultrastructure of neurons.

## Discussion

In this study, we introduce a protocol for the osmium maceration method that enables the direct 3D observation of membranous organelles in neurons via SEM without requiring time-consuming and labor-intensive reconstruction. To our knowledge, this is the first time the osmium maceration method has been described as a detailed, step-by-step protocol. The maceration method also provides insights into the spatial relationships among membranous structures, such as the Golgi apparatus, mitochondria, and ER (Koga and Ushiki 2006; Koga et al. 2017). Unlike conventional TEM, which yields only 2D information on subcellular structures, this method enables a more comprehensive understanding of the cellular architecture by allowing the 3D surface imaging of organelles.

A mixture of 0.5% glutaraldehyde and 0.5% paraformaldehyde is commonly used as a pre-fixative in the osmium maceration method (Tanaka and Mitsushima 1984). The concentration of this pre-fixative is lower than that used for conventional SEM specimen preparation (2% glutaraldehyde). In tissues treated with a highly concentrated fixative, specifically 2% glutaraldehyde, the cytoplasmic matrix is strongly fixed, which interferes with the osmium maceration process owing to the insufficient removal of the matrix from the cracked cell surfaces. Thus, a relatively dilute fixative composed of 0.5% glutaraldehyde and 0.5% paraformaldehyde is most effective for sufficiently removing the cytoplasmic matrix from the cracked cell surfaces during the maceration (Tanaka and Mitsushima 1984).

Under the conventional temperature condition of 20 °C, the osmium maceration procedure requires a considerable amount of time, typically several days, and the optimal duration for specimen preparation varies depending on the cell type (Koga et al. 2020). The maceration process requires 120 h to achieve optimal results for neurons of the spinal cord and trigeminal ganglion (Fig. 10). Therefore, the long duration of the maceration procedure is a drawback of this method. To improve the efficiency of the procedure, we recently established a rapid osmium maceration method, wherein an increase in the treatment temperature from the conventional 20 °C temperature to 30 or 40 °C markedly shortened the time required for maceration (Koga et al. 2020). Treatments at higher temperatures drastically reduced the optimal maceration duration to 30–40 h for various cell types, for instance, the optimal duration for motor neurons and trigeminal ganglion cells was 35 h (data not shown). Compared to conventional maceration at 20 °C, the rapid osmium maceration method reduces the maceration duration and offers greater stability and reproducibility.

When biological specimens with nonconductive properties are observed via SEM without conductive treatment, charging artifacts (caused by the accumulation of electrons within the specimens) can arise and disrupt the image quality. To enhance this process, conductive staining—a technique that involves embedding metals within tissue specimens—has been developed. Various conductive staining techniques have been reported; however, we prefer to use the “tannin–osmium method” (Murakami 1973) due to its simplicity and stability. In this method, an osmium solution is used as a conductive stain, while a tannic acid solution serves as a mordant to effectively facilitate heavy metal (i.e., osmium) binding to the specimen. The merits of conductive staining include the following: (1) reduced amount of metal coating; (2) enhanced conductivity and structural robustness of the specimen, leading to reduced deformation during dehydration and drying; and (3) reduction in specimen damage induced by electron-beam irradiation during SEM. Osmium-macerated tissues, which primarily comprise membrane components, are structurally fragile. Therefore, conductive staining was crucial for such specimens.

Biological specimens containing a large amount of water should be dried prior to SEM, as the vacuum environment within the column and specimen chamber leads to specimen deformation due to water evaporation. If specimens are dried without any prior treatment, simply through air-drying, the surface tension influences the drying process, resulting in severe tissue deformation and shrinkage. Therefore, specimens were dried under conditions free of surface tension. Two drying methods that avoid the effects of surface tension are commonly used, specifically, critical-point drying and freeze-drying. Both critical-point drying and freeze-drying effectively preserved the ultrastructure of the osmium-macerated specimens (Inoue 1992).

The conductivity of dried specimens can be enhanced by applying a thin metal coating to the surface. The combination of conductive staining and metal coating ensures the reliable and stable conductivity of the tissue specimens. Metal coating is a pivotal process that prevents the charging of artifacts and increases the efficiency of secondary electron generation during SEM. Furthermore, specimens coated with metals showed reduced thermal damage to the tissue caused by the electron beam irradiation during SEM. Metal targets for sputter coating include gold, platinum, and palladium. Metals with small grain sizes and high secondary electron yields are considered optimal for coating. The grain size decreases in the following order: gold, gold–palladium, platinum–palladium, and platinum. The choice of the metal target varies depending on the observation magnification; for the particularly high-magnification SEM of osmium-macerated tissues, platinum or platinum–palladium is recommended owing to the finer grain size.

Since osmium-macerated specimens are used to observe organelles, ultra-high-resolution SEM imaging is essential. The resolution of the scanning electron microscope is primarily determined by the electron gun (filament) and objective lens. The electron guns used in SEM are classified into three types, namely tungsten filaments, Schottky emitters, and field-emission (FE) emitters. Among these, systems equipped with Schottky or FE guns are considered high-resolution instruments, owing to their high electron beam brightness. Objective lenses are categorized into three types, out-lens, semi-in-lens, and in-lens. Among these, scanning electron microscopes equipped with semi-in-lens or in-lens systems are the most suitable for high-resolution observations. Conventional scanning electron microscopes with out-lens systems and tungsten filament thermal electron guns lack the resolution necessary to reliably visualize organelles in osmium-macerated specimens. The observation of intracellular organelles in osmium-macerated tissues requires scanning electron microscopes equipped with Schottky or FE electron guns, which provide the essential resolution for this purpose. Organelles can be visualized using out-lens SEM systems provided that the instrument is equipped with a Schottky or FE electron source. We usually examine osmium-macerated tissues using a semi-in-lens-type FE scanning electron microscope, which enables satisfactory imaging of the 3D ultrastructure of organelles (Fig. 10).

In the osmium maceration method, all procedures, including fixation, osmium maceration, conductive staining, dehydration, drying, mounting, and metal coating, are crucial for preparing optimal specimens for SEM observations. Recently, we developed novel SEM techniques based on the osmium maceration method to explore its further potential. Although the osmium maceration method is primarily applied to tissues and cells in vivo, we successfully expanded its applicability to cultured and free cells, such as blood cells, by incorporating agarose embedding into the maceration method (Koga et al. 2012). The osmium maceration method combined with agarose embedding is also expected to enable the observation of the membrane systems in cells that form organoids. In addition, by combining the Tokuyasu cryosectioning technique (Tokuyasu 1989) with the osmium maceration method, we successfully achieved correlative imaging between immunofluorescence and the 3D ultrastructural SEM images of organelles (Koga et al. 2015). In this study, we introduced the osmium maceration method using neurons and neural tissues as representative examples; however, this technique is applicable to neural cells and tissues as well as various other cell and tissue types. Therefore, we hope that the protocol for the osmium maceration method introduced in this paper will be referenced and will inspire its application to various tissues. Furthermore, we consider that future studies should focus on extending the application of the maceration method to pathological specimens. 

## Supplementary Information

Below is the link to the electronic supplementary material.

Supplementary file1 (PPTX 688 KB)

## Data Availability

All data generated or analyzed during this study are included in this published article and its supplementary information files.
