# Supplementary figures and images for: Direct imaging of the three-dimensional ultrastructure of neuronal organelles
Source: Anat Sci Int. 2025 Aug 5;100(4):598–613. doi: 10.1007/s12565-025-00888-5 (PMC12513917; doi:10.1007/s12565-025-00888-5)

## Slide 1
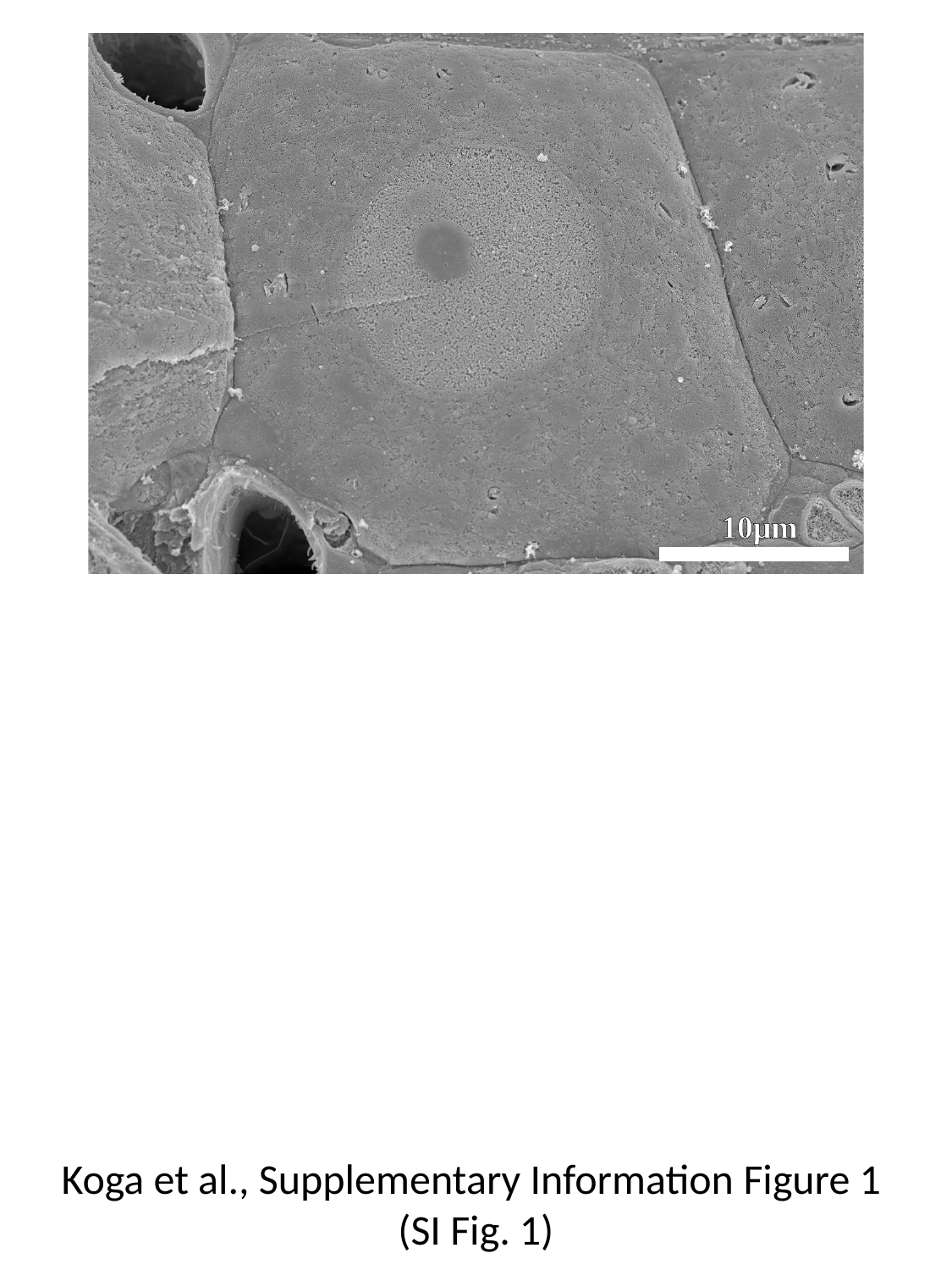

10µm
Koga et al., Supplementary Information Figure 1
(SI Fig. 1)

Supplement: Supplementary file 1 — Supplementary file1 (PPTX 688 KB) [file 12565_2025_888_MOESM1_ESM.pptx]
